# Ampullary carcinoma prognostic markers

**DOI:** 10.18632/oncotarget.27067

**Published:** 2019-07-16

**Authors:** Geraldine Perkins, Pierre Laurent-Puig, Julien Taieb

**Affiliations:** Sorbonne Paris – Cité, Paris Descartes University, Department of Gastroenterology and GI Oncology, Georges Pompidou European Hospital, Assistance Publique-Hôpitaux de Paris, Paris, France; Centre de Recherche UMR-S 1147, Médecine Personnalisée, Pharmacogénomique, Optimisation Thérapeutique, Institut National de la Santé et de la Recherche Médicale, Paris, France

**Keywords:** ampullary carcinoma, prognostic markers

The ampulla of Vater is located at the junction of the entry of both distal bile duct and the main pancreatic duct of Wirsung, into the second portion of duodenum. Neoplasms of this entity are rare, and usually classified in subtypes, mostly according from which part the tumor arises: pancreaticobiliary (PB), intestinal (INT), mixed, mucinous and poorly differentiated. In order to evaluate more accurately the prognosis of ampullary adenocarcinoma (AA), different tools have been investigated. For instance, histologic subtype has been reported as a prognostic factor, with PB subtype associated with a poorer outcome. Nevertheless, different studies have shown that clinicopathological scores/nomograms can evaluate prognosis independently of histologic subtype [1, 2]. Immunohistochemistry (IHC) has also been studied [3], with different marker panels, as well as molecular sequencing [4, 5], in order to better stratify AA, and refine prognostic evaluation.

We recently reported the results of a translational study from a retrospective AGEO cohort of nearly 100 AA patients, that evaluated and compared the prognostic values of several tools: the AGEO prognostic score (using age, general condition, tumour differentiation and TNM stage), the Ang et al-panel that combines 5 markers (CK7, CK20, CDX2, MUC1, MUC2), MUC5AC marker and 50 gene-panel [6]. Within these markers, the AGEO prognostic score and the tumor subtype were the only prognostic factors. Molecular alterations and MUC5AC had no impact on AA prognostication. Interestingly, we found similarities between INT-subtype and colorectal cancer on the one hand, and PB-subtype and pancreatic cancer on the other hand.

At the era of biomarkers, it is quite unusual to point out that clinicopathological scores have the most valuable impact in outcome evaluation of cancer patients. It may be explained by the weak power of statistical analysis due to rarety of the disease and the complexity of histopathological classification. This point is reinforced by a recently published work that showed the independent prognostic value of a clinicopathological nomogram using age, tumor grade and size, lymph/node ratio, extension range and histological type [2].

Moreover, we showed that pathological classification in AA allows identifying PB-subtype as a negative prognostic factor, as previously published. Interestingly, IHC classification and morphological classification were independent factors. The addition value of Ang et al. panel seems to mainly help in classification of undetermined samples. As the choice of chemotherapy in AA patients, even if no guidelines exist, is usually oriented by histologic subtype, a more accurate classification of doubtful cases seems important. In fact, many centers will prefer colon cancer chemotherapy regimens for intestinal AA and pancreatic chemotherapy regimens for PB AA.

Finally, even we didn’t find any prognostic value of molecular alterations, these features may help individual patients for precision medicine. In our series, we identified targetable alterations such as microsatellite instability, *ERBB2* amplification, suggesting a possible sensitivity to immune-check point inhibitor and anti-HER2 therapies respectively. Moreover, nearly half of tumors *RAS* wildtype, opening the possibility of testing anti-EGFR antibodies in clinical trials, at least for intestinal AA. A recent work, part of MSK-IMPACT project, identified in AA patients targetable somatic but also germline gene mutations, such as *BRCA2* and *ATM,* suggesting a potential subgroup that may respond to PARP-inhibitors [7]. Notably, even if patients with germline mutations had a family history of cancer, only half met criteria for germline genetic testing. Altogether, even not prognostic there may be still a room for wide molecular testing of AA tumors for theranostic purpose.
